# Dietary thiols in exercise: oxidative stress defence, exercise performance, and adaptation

**DOI:** 10.1186/s12970-017-0168-9

**Published:** 2017-04-27

**Authors:** Yanita McLeay, Stephen Stannard, Stuart Houltham, Carlene Starck

**Affiliations:** 1 School of Sport and Exercise, Massey University, Private Bag 11-222, Palmerston North, New Zealand; 2 Massey Institute of Food Science and Technology, Massey University, Palmerston North, New Zealand

**Keywords:** Endurance, Thiol, Antioxidant

## Abstract

Endurance athletes are susceptible to cellular damage initiated by excessive levels of aerobic exercise-produced reactive oxygen species (ROS). Whilst ROS can contribute to the onset of fatigue, there is increasing evidence that they play a crucial role in exercise adaptations. The use of antioxidant supplements such as vitamin C and E in athletes is common; however, their ability to enhance performance and facilitate recovery is controversial, with many studies suggesting a blunting of training adaptations with supplementation. The up-regulation of endogenous antioxidant systems brought about by exercise training allows for greater tolerance to subsequent ROS, thus, athletes may benefit from increasing these systems through dietary thiol donors. Recent work has shown supplementation with a cysteine donor (N-acetylcysteine; NAC) improves antioxidant capacity by augmenting glutathione levels and reducing markers of oxidative stress, as well as ergogenic potential through association with delayed fatigue in numerous experimental models. However, the use of this, and other thiol donors may have adverse physiological effects. A recent discovery for the use of a thiol donor food source, keratin, to potentially enhance endogenous antioxidants may have important implications for endurance athletes hoping to enhance performance and recovery without blunting training adaptations.

## Background

It is well-established that muscular work is associated with oxidative stress and that prolonged or high-intensity exercise results in oxidative damage to macromolecules in both blood and skeletal muscle [1–3]. This exercise-induced oxidative damage can impair physical performance via various mechanisms relating to compromised myocellular structure and function. Furthermore, chronic oxidative stress in athletes, often brought about by overtraining, has been linked to chronic fatigue [4], longer term performance decrements [5], muscle atrophy [6], and illness [7]. Exogenous and endogenous antioxidants reduce oxidative stress, thus it is not surprising that dietary antioxidants are a popular supplement for athletes in an attempt to enhance recovery, optimise performance, and reduce the oxidant load to maintain long-term health.

The two groups of biologically active molecules that fall under the umbrella term ‘free radicals’ include reactive oxygen species (ROS) and reactive nitrogen species (RNS). During exercise, oxygen consumption can increase as much as 100-fold [8] and is associated with rapid increases in ROS production and accumulation. This constant production of free radicals by skeletal muscle requires the buffering capacity of an endogenous defense system, and a multitude of mechanisms have evolved to detect and respond to elevated oxidant production. The action of these systems determines the overall endogenous antioxidant capacity, and if this capacity is exceeded, oxidative stress ensues, potentially resulting in detrimental oxidation of cell membranes, functional proteins, and DNA [9–11].

Paradoxically, the redox activities of ROS and RNS play critical roles in cell signaling and exercise adaptation, a phenomenon summarized by the concept of hormesis, where low levels of stress promote adaptation to and thus protection from, subsequent stress [12]. Various exercise studies have observed improved endogenous defence in rodents following training [13–17], along with reduced ROS production at the same absolute exercise workload during following training [18]. However, at very high concentrations, ROS can move beyond being advantageous, and have detrimental effects on performance. During heavy endurance training, endogenous antioxidant capacity cannot meet the increasingly high ROS generation, resulting in a state of oxidative stress and subsequent cellular damage [19]. Dietary supplementation with antioxidants in this situation may be beneficial; however, the exact ‘balance’ of (exogenous) antioxidant supplementation to ROS levels that may enhance performance without blunting adaptive pathways is currently unknown. Commonly supplemented antioxidants include the well-known vitamins such as C and E, and in recent times, research concerning the effects of these on exercise performance in humans has grown considerably, producing equivocal results [20–24]. While acute administration in humans may potentially enhance performance [25], the majority of studies suggest no benefit [23, 26–30]. Furthermore, there is concern that chronic supplementation may blunt the free radical-driven adaptive effects of training by interfering with the essential endogenous antioxidant response to ROS [31, 32]. More recently, the thiol donors’ glutathione, cysteine, and taurine, have generated interest for the improvement of exercise recovery and performance, with their potential to boost endogenous antioxidant defence in a way that generalized antioxidants cannot. However, the use of these is limited by poor supplement bioavailability and several side effects [33, 34]. This has created a niche for the advent of palatable, digestible, and tolerated dietary thiol sources. In this review we focus on the emergence of thiols as antioxidant supplements for the endurance athlete, the current research surrounding their ergogenic potential and benefits, and recent efforts to produce a high-cysteine food source.

## Antioxidant supplementation, exercise performance, and adaptation

While research into the effect of common dietary antioxidants on acutely reducing free-radical load is well documented in humans, evidence of any exercise performance or adaptation benefits are equivocal [20–23, 30, 31, 35–38]. Few studies have observed performance benefits as a result of supplementing with specific dietary antioxidants [37] and many well controlled studies in this area have found no performance benefit [21–23, 39–42]. It has been suggested that antioxidant supplementation may only improve performance when endogenous levels are already depleted, and after reaching normal concentrations, no further benefit is seen [35]. Similarly, antioxidant supplementation does not appear to benefit recovery time following acute exercise [43–46], with only a limited number of studies showing minor recovery benefits [30, 47]. On the other hand, there is an increasing body of work that has shown that ingestion of specific foods, high in antioxidant compounds can accelerate recovery [44, 48, 49]. Both cherries [44, 48] and blueberries [49] seem to expedite the recovery of muscular force following strenuous eccentric work. However, whether this is due to the antioxidant capabilities of the foods, or the polyphenolics is a point of contention. Our previous work [49] seems to infer the latter.

A number of published studies to date indicate that supplementation with common dietary antioxidants may actually be detrimental to athletic performance by blunting the adaptive effect of exercise training [31, 50–52]. These adaptations, including upregulated endogenous defense, muscle protein synthesis, and mitochondrial biogenesis, are important for improved exercise capacity and recovery. Dietary antioxidants such as vitamins C and E are generally required in very small amounts, working with endogenous antioxidants to maintain or re-establish redox homeostasis [53]. However, these general antioxidants non-specifically scavenge all free radicals, regardless of their source; hence increased intake may affect various pathways including cellular signaling pathways that are important for exercise adaptation. In contrast, endogenous antioxidant systems are a complex and compartmentalized network that involve controlled and localized production of specific ROS [54]. These ROS are uniquely balanced by endogenous antioxidants to levels where cellular signaling can still be carried out. In athletes, disruption of this system by over-supplementation with generalized exogenous antioxidants can result in blunted signaling pathways [31, 38, 50]. This in turn can compromise recovery and attenuate exercise adaptations, negatively affecting performance.

In addition to their effect on adaptive pathways, at high levels, exogenous antioxidants can promote oxidation, contributing to acute decrements in exercise performance. For example, vitamin C can react with metal ions released from exercise-induced tissue damage, giving rise to harmful hydroxyl radicals [55], and vitamin E can become a free radical itself when it reacts with a free radical [56], damaging lipid membranes if it is not converted back to its reduced form. Following acute eccentric induced muscle injury in young men, Childs et al. [57] found increased oxidative stress and tissue damage supplementation with vitamin C and a cysteine derivative n-acytylcysteine (NAC), compared to a placebo. Similarly, in ultra-endurance (Ironman™) competitors, vitamin C or E supplementation prior to racing significantly elevated markers of oxidative stress and showed significant decreases in antioxidant enzyme activity post- race, compared with no supplementation [58]. For a more comprehensive review on the various dietary antioxidants that can form a pro-oxidant state, please refer to Yordi et al. [59].

In contrast to exogenous antioxidants, high levels of endogenous antioxidants do not appear to contribute to oxidative stress. Rather, it is depleted levels that can give rise to an oxidant state [60]. It could therefore be suggested that consuming dietary sources of endogenous antioxidant precursors, such as thiol donors, may enhance exercise performance and recovery without the negative effects associated with exogenous antioxidant supplementation.

## Thiols

### Antioxidant capacity

**Fig. 1:**
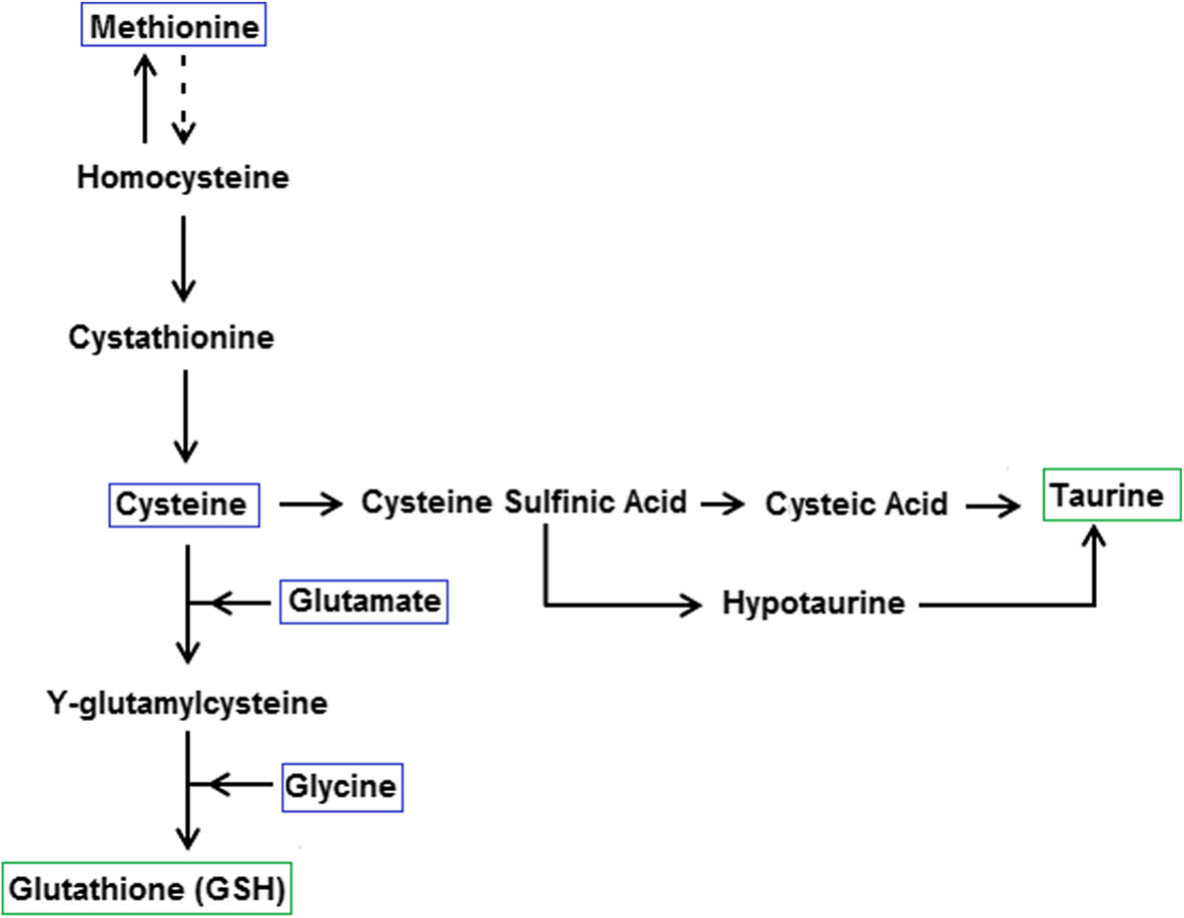
Summary of thiol metabolism and resulting thiol antioxidants. Methionine is metabolized to cysteine which further forms endogenous antioxidants taurine and glutathione via two distinct pathways. Supplementing with taurine and/or cysteic acid may ‘spare’ cysteine, upregulating GSH synthesis, thus further boosting endogenous antioxidant defence

Thiols, molecules that contain a sulfhydyl (SH) side chain group, act as antioxidants, stabilizing free radicals by accepting their unpaired electron. Methionine and cysteine are two key dietary thiol amino acids that metabolize to the powerful and arguably most important endogenous antioxidant, glutathione (GSH). Adequate levels of GSH are crucial in maintaining reduction-oxidation (redox) balance within body tissues with the ratio of reduced to oxidized glutathione (GSH/GSSG) being a primary indicator of redox state. A higher ratio of GSH to GSSG suggests a reductive environment where ROS levels are kept at homeostatic levels, whereas a low GSH to GSSG ratio is indicative of oxidative stress [61]. It is cysteine that gives GSH its antioxidant activity; as cysteine is also rate limiting to its formation, dietary cysteine, or its precursor amino acid methionine, is crucial for maintaining endogenous antioxidant defense. In addition to GSH, dietary thiols have the ability to increase levels of taurine, another powerful thiol antioxidant, via the cysteine sulfinic acid pathway (Fig. 1).

Various studies using thiol donor supplementation have shown positive effects on the up-regulation of endogenous antioxidants and antioxidant enzyme activity. For example, administration of the cysteine-donor N-acetyl cysteine (NAC) in humans significantly increases blood [62–64] and muscle [65, 66] GSH levels, and in vitro studies show methionine supplementation to have beneficial effects on GSH hepatocyte concentrations [67]. Furthermore, NAC has been shown to increase taurine levels in skeletal muscle [68].

### Exercise performance

While it appears that excessive intakes of common dietary antioxidants may contribute to oxidative stress [56, 69], there is no published literature to suggest high levels of endogenous thiol antioxidants to have the same effect. Exercise appears to significantly alter GSH and taurine tissue levels, and supplementing with thiol donors has been shown to attenuate this [70]. Reduced markers of oxidative damage have been seen post-exercise with thiol supplementation [71], suggestive of reduced ROS levels. Thus, in contrast to the well-researched vitamins C and E, several human studies have suggested that the observed ergogenic effect of thiol donors such as NAC is due to improved endogenous defense systems [72, 73].

Perhaps due to potential toxicity associated with methionine supplementation, to our knowledge no published studies have looked into the use of methionine on exercise performance in either humans or animals. Similarly, as an isolated supplement, L-cysteine has been shown to elicit toxic effects in several animal studies [74–76]. Because of this, the majority of studies looking at cysteine supplementation on endogenous antioxidant up-regulation use NAC, which endogenously cleaves cystine into two cysteine molecules [77]. Additionally, studies that use exogenous forms of GSH are not common as it is primarily degraded to its constituent amino acids [78]. Exogenous taurine supplementation however, has shown some promising results on increasing time to fatigue during exhaustive exercise by up to 50% in rodents, and time to VO_2max_ in men [79–81]. Therefore, increasing GSH and taurine levels within the body via thiol donors may benefit athletes.

In rats, NAC infusion at 150 mg.kg^−1^ was found to increase tolerance to respiratory loading, while also attenuating a decrease in diaphragm GSH [82]. Similarly in humans, the same infusion rate of NAC during electrical stimulation of the ankle dorsiflexors was shown to increase force output by 15% compared to placebo [83]. Furthermore, Medved and colleagues [65] found that infusing NAC (150 mg.kg^−1^) into endurance trained men prior to a fatiguing cycling protocol gave rise to a 26.3% increase in time to exhaustion at 92% VO_2_max compared to placebo; although in other research from the same group [73], cycling time to fatigue was not affected by an infusion of 125 mg.kg^−1^, despite an attenuation in the reduction of GSH. In contrast to these studies, Matuszaczak et al. [84] found an infusion of 150 mg.kg^−1^ to have no effect on force production during sustained maximal handgrip contractions compared to placebo. It did however inhibit GSH oxidation.

A role for taurine has been implicated in virtually every body tissue, with the highest concentrations found within skeletal and cardiac muscle [85]. Maintaining adequate taurine is important for endurance athletes, as taurine also plays crucial roles in muscle contraction and relaxation [86], lipid metabolism [87], and potentially muscle protein synthesis [88]. Furthermore, a possible ‘sparing’ of GSH has been observed with taurine supplementation [89], suggesting indirect redox-balancing roles. While several earlier studies, using taurine-containing energy drinks, observed a combination of taurine and caffeine to enhance exercise performance above that of caffeine alone [90, 91], it is only more recently that isolated taurine has been used in exercise research. Several studies have shown that two weeks of taurine supplementation in rats elicits 50% [81, 92] and 34% [79] greater time to exhaustion during treadmill running compared to non-supplemented (control) animals. The impact of taurine supplementation on human exercise performance is less clear. In recreationally trained men, taurine supplementation prior to cycling to VO_2max_ reduced oxidative stress markers in plasma post-cycle, and also significantly increased time to exhaustion at VO_2max_, and maximal workload [80]. Similarly, in trained runners taurine ingestion prior to a 3-km time trial significantly improved performance [93]. In contrast however, Rutherford et al. [94] found acute ingestion of taurine to have no effect on time-trial performance in well-trained cyclists, although it did appear to significantly increase fat oxidation during submaximal cycling at the same absolute work rate.

Overall, the effect of thiol donors on human performance appear to vary, probably due to vast methodological differences including exercise protocol, dosage amount, length of time, and subject training status. However, if the provision of precursors for endogenous antioxidants, such as NAC for GSH and/or taurine, enables the body to regulate to optimal levels its own antioxidant defence mechanism, this may be preferable to the use of general exogenous antioxidants such as vitamins C and E.

### Exercise and adaptation

In addition to performance, the ability to improve physical capacity over time is of utmost importance for athletes. The human body has the ability to adapt to exercise stressors, allowing for a greater tolerance to a subsequent similar workload, and relies on various ROS-mediated signaling pathways to do so. At low levels, exercise-induced ROS play a critical role in skeletal muscle adaptation [95] by up-regulating various cytokines and protein kinases such as tumor necrosis factor alpha (TNF-α) [96] and mitogen-activated protein kinases (MAPK) [97] that work to signal increases in mitochondrial density and number, endogenous antioxidants, and assist in muscle protein synthesis [98, 99]. Certain studies have observed up-regulated endogenous antioxidants and antioxidant enzymes, specifically superoxide dismutase (SOD) and glutathione peroxidase (GPx) following endurance training [18, 100], and well trained athletes appear to have higher base-line levels of these enzymes, as well as GSH [101]. Additionally, while muscle protein breakdown is stimulated during exercise, protein synthesis is increased during recovery through ROS signaling [102]. The use of common antioxidants vitamins C and E prior to exercise have been shown to mitigate ROS signaling pathways, resulting in the blunting of such adaptations [31, 36, 50–52].

While exogenous antioxidants may attenuate training adaptations, there is no literature to suggest that increasing endogenous antioxidants has this effect. Rather, a recent study [103] in cyclists undergoing strenuous physical training observed improved physiological adaptation with a week of oral NAC while several other studies [72, 104] have found increased signaling cytokines such as TNF-α with NAC supplementation compared to a placebo, despite improved performance. This suggests that NAC may contribute to exercise adaptation by increasing levels of the various signaling molecules. As opposed to oral supplementation however, studies using direct infusion of NAC appear to attenuate the increase in these signaling molecules [105, 106]. Thus it is important to consider the method of administration when analyzing effect. Whilst NAC appears to upregulate endogenous antioxidants in those who have depleted levels, in healthy individuals, high doses may have pro-oxidant effects [107–109]. Furthermore, in some individuals, oral [110] and systemically administered [83] NAC can be poorly tolerated. Little research exists on taurine’s effect on exercise adaptions; however, taurine appears to have potent cytoprotective roles in skeletal muscle [88, 111]. Various studies have shown taurine depletion or attenuation of its transport to reduce muscle function and increase atrophy [112]. In addition, several studies have observed a reduction in eccentric-induced muscle damage [113] and oxidative stress [113, 114] following taurine supplementation, suggesting its potential role in recovery via reducing initial tissue damage.

Additional research into the effects of long term use of thiol donors is necessary; however, early observations suggest that they may be useful as ergogenic aids for endurance athletes without attenuating useful adaptations.

## A future of thiols as ergogenic aids

Despite their potential benefits, the use of supplementary thiol donors to improve endogenous antioxidant status may not necessarily be the ideal way to enhance performance and recovery. As previously mentioned, high intakes of NAC in healthy individuals may have pro-oxidant effects [107–109], and both oral [110] and systemically administered [83] NAC can be poorly tolerated. Similarly, while methionine supplementation has been shown to increase GSH levels in those with sub-optimal levels [115, 116], excess intakes in healthy individuals may increase homocysteine levels which has been shown to contribute to cardiovascular disease and several mental health disorders [117, 118]. Therefore, alternative dietary sources of thiols may provide a safer option for athletes and others.

### Food-based thiol donors

Keratin is found in tissues including hair, skin, nails, and feathers, and is comprised of cysteine-based disulfide bonds. While necessary for structural purposes, these strong linkages between cysteine residues render keratin indigestible to humans and thus unavailable for absorption. However, hydrolysed keratin, through either acid or alkaline hydrolysis vastly improves digestibility [119] and sets this protein up as a potential thiol source. High in cysteic acid, a metabolite of cysteine, keratin hydrolysate may directly increase taurine levels; indeed, a recent study in rats [120] observed increased liver taurine following four weeks of keratin supplementation, along with the maintenance of GSH levels, suggestive of a sparing effect. A later study tested the same keratin protein in humans over two weeks using a ramped dose protocol. Starting at 10 g of keratin/day (10.g.d^−1^) for three days, the supplementation period finished with an intake of 40 g.d^−1^; a level at which there were still no adverse physiological effects reported [119]. These two studies may have implications for the use of keratin-based thiol supplementation in athletes; however, research into this area is scarce.

## Conclusions

Endurance athletes are susceptible to cellular damage initiated by excessive levels of aerobic exercise-produced ROS. Whilst ROS can contribute to the onset of fatigue, there is increasing evidence that they play a crucial role in exercise adaptations. The use of general antioxidant supplements such as vitamin C and E in athletes is common; however, their ability to enhance performance and facilitate recovery is controversial, with many studies suggesting a blunting of training adaptations with chronic supplementation. However, as the up-regulation of endogenous antioxidant systems are brought about by exercise training, athletes may benefit from increasing these systems through dietary thiol donors. While the thiol donors methionine and NAC may increase endogenous antioxidants and antioxidant enzymes, there can be adverse effects associated with their use. Thus, the discovery for the use of hydrolysed keratin to potentially enhance endogenous GSH and taurine may have important implications for athletes hoping to enhance performance and recovery without blunting training adaptation.

## Abbreviations

GPx: Glutathione peroxidase
GSH: Glutathione (reduced)
GSSG: Glutathione (oxidized)
H_2_O_2_: Hydrogen peroxide
MAPK: Mitogen-activated protein kinases
NAC: N-acetyl cysteine
RNS: Reactive nitrogen species
ROS: Reactive oxygen species
SOD: Superoxide dismutase
TNF-α: Tumor necrosis factor-alpha
